# Healthcare Utilization and Costs Reduction after Radiofrequency Ablation for Atrial Fibrillation in the Brazilian Private Healthcare System

**DOI:** 10.5935/abc.20190148

**Published:** 2019-08

**Authors:** Alvaro Sarabanda

**Affiliations:** 1 Instituto de Cardiologia do Distrito Federal, Brasília, DF - Brazil

**Keywords:** Arrhythmias, Cardiac, Atrial Fibrillation, Radiofrequency Ablation/methods, Arrhytmias/drug therapy

Atrial fibrillation (AF) is the most common cardiac arrhythmia, constituting an important public health problem and leading to excessive spending on health care worldwide.^1,2^ It has important repercussions in clinical practice, associated with an increased risk of stroke, development of heart failure, cognitive alterations, decreased quality of life and increased risk of death.^1^

It is estimated that in the American adult population the incidence of AF will increase from 1.2 million cases per year in 2010 to 2.6 million in 2030 and, in the same period, its prevalence will increase from 5.2 million to 12 million people.^3^ In Brazil, AF estimates are less accurate. However, a recent epidemiological study with the Brazilian population reported a prevalence of AF of about 1.8% in the general population.^4^ However, considering the aging of the population in middle-income countries such as Brazil, the prevalence of AF in our country is likely to increase in the near future.^5^

A recent study^2^ reported that in 2010 the total annual cost for treatment of AF was about 26 billion dollars in the United States and, due to the epidemic growth of this arrhythmia, the cost of its treatment should increase substantially in the coming years all around the world. Much of this cost is due to recurrent hospitalizations, emergency room visits, and outpatient follow-up. In this sense, an immediate evaluation of the health costs used in the treatment of this arrhythmia becomes a priority in our environment.

About 20 years ago, percutaneous radiofrequency ablation of the pulmonary veins (PVs) was described by Haissaguerre et al.^1,6^ as an effective and curative technique for the treatment of paroxysmal AF. Subsequently, the ablation procedure of the PVs was progressively modified, evolving to the current predominant technique of enlarged antral circumferential ablation of PVs (an enlarged area of 1 to 2 cm of the PV ostia) in order to modify the arrhythmogenic substrate responsible for the triggering and maintenance of AF.^1^

In this context, it has consistently been shown in several randomized clinical studies that percutaneous ablation of AF reduces the recurrence of this arrhythmia, greatly improving patients' quality of life^7,8^ and cardiac mortality in patients with left ventricular dysfunction,^9^ as compared to antiarrhythmic therapy. Additionally, nonrandomized clinical studies have reported that AF ablation also reduces the risk of stroke.^10^

Thus, it is possible to speculate that patients with AF undergoing catheter ablation should present a significant reduction in the use of health care and its related costs, both due to the decrease in hospitalizations, as well as the reduction of emergency room visits and outpatient follow-up.^11^

In this issue of *Arquivos Brasileiros de Cardiologia*, Saad et al.^11^ report their findings on the use of health care, including outpatient and hospital care, as well as their costs, in a retrospective cohort of Brazilian private health care patients, before and after catheter ablation for AF. Between January 2014 and December 2015, 83 patients undergoing AF ablation were identified as the study cohort, and their data were analyzed for the mean period of 14 months prior to ablation and 10 months after the procedure.

In the study under analysis, in agreement with the world literature, there was a significant reduction of the health costs for the treatment of AF after catheter ablation.^12,13^ The 1-year AF recurrence-free rate was 86%. As a result, the median of the total monthly costs had a reduction of 68.5% (p < 0.001) after ablation. Ambulatory and emergency costs were also reduced by 48.8% and 100%, respectively, (p < 0.001 for both variables) after AF ablation.

However, as pointed out by the authors, the study has several limitations. The data set used for all analyzes was based on patient billing information, which may have overestimated the success rate of AF ablation, since AF recurrence was based only on the use of health resources (use of antiarrhythmic drugs in the emergency room, cardioversion or repetition of procedures), or indirectly, in the purchase of antiarrhythmic drugs in pharmacies. The use of an administrative database carries the risk of bias, with the problems associated with the lack of individual clinical information of the patients, as well as the retrospective design of the study. In this sense, the results of this study can not be applied to all subgroups of patients with AF (for example, newly detected AF, persistent or long-standing persistent AF), since the patients' AF characteristics were not reported. Finally, the sample size was small and the analysis of the possible predictors of the greatest cost reduction after ablation was probably poor.

Finally, the present study has the merit of demonstrating that, in relatively young patients with few comorbidities and in need of increased health care for the treatment of AF, catheter ablation of this arrhythmia can significantly reduce the costs of outpatient and hospital care in the medium term follow-up after ablation.
